# Upregulation of SATB1 is associated with the development and progression of glioma

**DOI:** 10.1186/1479-5876-10-149

**Published:** 2012-07-28

**Authors:** Sheng-Hua Chu, Yan-Bin Ma, Dong-Fu Feng, Hong Zhang, Zhi-An Zhu, Zhi-Qiang Li, Pu-Cha Jiang

**Affiliations:** 1 Department of Neurosurgery, NO.3 People's Hospital Affiliated to Shanghai Jiao Tong University School of Medicine, 280 Mo He Road, Bao Shan District, Shanghai, 201900, China; 2 Department of Neurosurgery, Zhongnan Hospital of Wuhan University, Wuhan, 430071, China

## Abstract

**Background:**

Special AT-rich sequence-binding protein-1 (SATB1) has been reported to be expressed in several human cancers and may have malignant potential. This study was aimed at investigating the expression and potential role of SATB1 in human glioma.

**Method:**

The relationship between SATB1 expression, clinicopathological parameters, Ki67 expression and MGMT promoter methylation status was evaluated, and the prognostic value of SATB1 expression in patients with gliomas was analyzed. SATB1-specific shRNA sequences were synthesized, and U251 cells were transfected with SATB1 RNAi plasmids. Expression of SATB1 mRNA and protein was investigated by RT-PCR and immunofluoresence staining and western blotting. The expression of c-Met, SLC22A18, caspase-3 and bcl-2 protein was determined by western blotting. U251 cell growth and adherence was detected by methyl thiazole tetrazolium assay. The apoptosis of U251 cells was examined with a flow cytometer. The adherence, invasion, and *in vitro* angiogenesis assays of U251 cells were done. The growth and angiogenesis of SATB1 low expressing U251 cells was measured in an *in vivo* xenograft model.

**Results:**

Of 70 tumors, 44 (62.9%) were positive for SATB1 expression. SATB1 expression was significantly associated with a high histological grade and with poor survival in univariate and multivariate analyses. SATB1 expression was also positively correlated with Ki67 expression but negatively with MGMT promoter methylation in glioma tissues. SATB1 shRNA expression vectors could efficiently induce the expression of SLC22A18 protein, increase the caspase-3 protein, inhibit the expression of SATB1, c-Met and bcl-2 protein, the growth, invasion, metastasis and angiogenesis of U251 cells, and induce apoptosis *in vitro*. Furthermore, the tumor growth of U251 cells expressing SATB1 shRNA were inhibited *in vivo*, and immunohistochemical analyses of tumor sections revealed a decreased vessel density in the animals where shRNA against SATB1 were expressed.

**Conclusions:**

SATB1 may have an important role as a positive regulator of glioma development and progression, and that SATB1 might be a useful molecular marker for predicting the prognosis of glioma.

## Background

Gliomas are a major class of human intrinsic brain tumors, which includes well differentiated low grade astrocytomas, anaplastic astrocytomas and glioblastoma multiforme, the most malignant brain tumor of adulthood. Although resection remains the most effective treatment for glioma, the high rate of postoperative recurrence inevitably leads to a poor clinical outcome [1,2]. An understanding of the genetic background and molecular pathogenic processes involved in the tumorigenesis of glioma is therefore critical for the development of rational, targeted therapies [3].

Special AT-rich sequence-binding protein 1 (SATB1) is a cell type-specific nuclear matrix attachment region (MAR)-binding protein that links specific DNA elements to its cage-like network [4], which is predominantly expressed in thymocytes [5]. It facilitates formation of an open chromatin structure and participates in the regulation of hundreds of genes. In recent years, a number of studies have suggested that it plays major roles in T-cell development, early erythroid differentiation, homeostasis and response to physiological stimuli [6-8]. In addition to discoveries of these physiological roles, SATB1 has recently attracted considerable attention due to its high expression in tumor tissues of a variety of malignancies, such as breast cancer [9], lymphoma [10], gastric cancer [11], colorectal cancer and laryngeal cancer [12,13], which suggest a crucial role in promoting tumor growth, invasion and metastasis, and may also have a potential value of being a candidate for cancer therapy [14]. In the current study, we sought to determine the expression and functional role of SATB1 in gliomas, in order to define the relationship between SATB1, tumor behavior and prognosis.

## Methods

### Patients and specimens

Seventy surgically resected human glioma specimens were collected at the Department of Neurosurgery, Zhongnan Hospital of Wuhan University between 2003 and 2005 and the NO.3 People's Hospital Affiliated to Shanghai Jiao Tong University School of Medicine between 2005 and 2006. Informed patient consent and prior approval from the Zhongnan Hospital of Wuhan University and NO.3 People's Hospital Affiliated to Shanghai Jiao Tong University School of Medicine Ethics Committees (Ethic approval ZNHWHU0388, NTPHSHJTUSM045) was obtained before the clinical materials were used for research purposes. Tissue from three normal brains was obtained from individuals who had died in traffic accidents without any prior pathologically detectable condition. All experiments on humans in the present study were performed in compliance with the Helsinki Declaration. The study group consisted of 54 men and 16 women with a median age of 45years (range, 1776years). None of these patients had received radiotherapy or chemotherapy prior to surgery. All tumor specimens were pathologically diagnosed as glioma. All samples were divided into two subgroups according to histological types: low grade gliomas (WHO grades I and II) and high grade gliomas (grades III and IV). Forty-five patients received focal fractionated radiotherapy; and 42 patients received postoperative chemotherapy. Postoperative chemotherapy was administered with temozolomide (TMZ) or teniposide (VM-26) together with semustine (methyl-[N-[2-chloroethyl]-N0-[4-methylcyclohexyl]- N-nitrosourea] [Me-CCNU]). Of these 42 patients, 40 also chose to undergo concomitant focal fractionated radiotherapy. All specimens were stored at 80C until analysis.

### Cell culture

ECV304 cells and human glioma cell line U251 (Wuhan University of China) were cultured in RPMI-1640 (Gibco Life Technologies, Paisley, Scotland, UK) supplemented with 10% fetal bovine serum 100g/ml penicillin, and 100g/ml streptomycin. Routine testing confirmed that the cells were free of *Mycoplasma* and viral contaminants during the entire study period.

### Knock down SATB1 by RNAi in U251 cells

SATB1-specific shRNA sequences were synthesized according to the one used in Han *et al.*[15]*.* and inserted into the pGCsi-H1/Neo/GFP/siNEGative vector (Genscript), which coexpresses GFP to allow identification of transfection efficiency. The SATB1 shRNA sequence was: SATB1-shRNA 5'-GTCCACCTTGTCTTCTCTC-3'. The non-specific shRNA sequence was: control-shRNA-GFP 5'-ACGTGACACGTTCGGAGAA-3' [16]. U251 cells were transiently transfected with SATB1 RNAi plasmids or control plasmids using an electroporator.

### Immunohistochemical analysis

Antigen retrieval was performed in boiling citrate buffer for 15 minutes. Peroxide blocking was performed with 0.3% peroxide in absolute methanol. The slides were then incubated with anti-SATB1 polyclonal antibody (diluted 1:100; Sigma, St Louis, MO) or mouse anti-PCNA monoclonal antibody (diluted 1:100; Santa Cruz) or anti-Ki67 (diluted 1:20; clone MIB-1, Dako, Denmark) at 4C overnight and washed twice with PBS before being incubated with the secondary antibody (Santa Cruz, CA) at room temperature for 30 mintes. After washing, sections were incubated with immunoglobulins conjugated with horseradish peroxidase (HRP). Finally, the reaction was developed with 3, 3'-diaminobenzidine substrate. Tissue sections were counterstained with hematoxylin or methyl green [17]. Immunohistochemical staining for CD34 and microvessel counting of CD34-positive vessels were performed as described previously [18].

The total SATB1 immunostaining score was calculated as the sum of the percentage positivity of stained tumor cells and the staining intensity scores. The percentage positivity was scored as follows: 0 (< 5%, negative); 1 (5%-25%, sporadic); 2 (25%-50%, focal); 3 (> 50%, diffuse). The staining intensity was scored as follows: 0 (no staining); 1 (weakly stained); 2 (moderately stained); 3 (strongly stained). Both the percentage positivity of cells and the staining intensity were assessed under double-blind conditions. The final SATB1 expression score ranged from 0 to 9 and was calculated as the percentage positivity scorestaining intensity score. The SATB1 expression level was defined as follows: - (score 01); + (score 23); ++ (score 46); +++ (score>6). The Ki67 index was calculated as the percentage of Ki67-positive cells in five independent high-magnification ( 200) fields per section [19,20].

### DNA extraction and MSP

Briefly, genomic DNA was extracted from tumor tissues by the digestion with proteinase K using the Genomic DNA Purification Kit (Gentra Systems, Minneapolis, MN, USA) and 1g genomic DNA was treated with the Chemicon CpG WIZ DNA Modification Kit (Chemicon International, Temecula, CA, USA) to convert unmethylated cytosines to uracil, leaving methylated cytosines unchanged. The modified DNA was diluted in TE buffer. O(6) -methylguanine-DNA-methyltransferase (MGMT) promoter methylation analysis was performed by PCR, using bisulfite-treated DNA as template, with specific primers for the methylated (unmodified by bisulfite treatment) and unmethylated (bisulfite modified) gene sequences using the MSP method.4 The MGMT primer sequences for the unmethylated reaction (UMS sense 5'-TTTGTGTTTTGATGTTTGTAGGTTTTTGT-3' and UMAS antisense 5'-AACTCCACACTCTTCCAAAAACAAAACA-3) were designed to amplify a 93bp product [21]. The MGMT primer sequences for the methylated reaction (MS sense 5'-TTTCGACGTTCGTAGGTTTTC GC-3' and MAS antisense 5'-GCACTCTTCCGAAAACGAAACG-3') were designed to amplify a 81bp product [21]. The results were confirmed by repeating the bisulfite treatment and MSP assays for all samples.

### Western blotting analysis

Untransfected U251, control-shRNA-GFP U251 or SATB1-shRNA U251 cells were washed in ice-cold PBS and lysed in buffer using standard methods [22]. The frozen samples of glioma and normal brain tissues were homogenized in a RIPA lysis buffer. Lysates were cleared by centrifugation (14,000rpm) at 4C for 30 minutes. Protein samples (approximately 40g) were separated by SDS-PAGE (15% gel), transferred to PVDF membrane and non-specific binding sites blocked by incubation in 5% non-fat milk for 60 minutes. Membranes were incubated overnight at 4C with polyclonal anti-SATB1 primary antibody (1:200 dilution; Sigma, St Louis, MO) or anti-c-Met antibody (1:400 dilution; Santa Cruz, CA) or anti-SLC22A18 antibody (1:1,000 dilution; Santa Cruz, CA) or anti-caspase-3 antibody (1:500 dilution; Dako, Glostrap, Denmark) or anti-bcl-2 antibody (1:300 dilution; Dako, Glostrap, Denmark). The membrane was then washed three times with TBST for 10 minutes and probed with HRP-conjugated secondary antibody (at 1:2,000 dilution; Dako, Glostrap, Denmark) for 30 minutes at room temperature. After being washed three times, the membrane was developed using an enhanced chemiluminescence system (ECL, Pierce).

### Total RNA isolation and reverse-transcriptase polymerase chain reaction

Total RNA was extracted from glioma tissues, normal brains, and U251 cells, using TRIzol (Invitrogen, Carlsbad, CA) following the manufacturer's instructions. The RT reaction was performed on 2g of total RNA using the SuperScript II First-Strand Synthesis and an oligo(dT) primer (Invitrogen). The SATB1 primer sequences and RT-PCR conditions were as previously described (forward primer 5'-CATTCAAGCTCCTTTCCCTTTC-3' and reverse primer 5'-TGGGCTCGTATC AACACCTATC-3') [23]. The housekeeping gene *GAPDH* was used as an internal control for the RT reaction (forward primer 5'-TGGGGAAGGTGAAGGTCG-3' and reverse primer 5'-CTGGAAGATGGTGATGGGA-3'). PCR was performed over 35 cycles at 94C for 1 minute, at 62C for 1 minute, and 72C for 1 minute followed by a final extension at 72C for 5 minutes and the PCR products were analyzed using 2% agarose gels.

### Immunofluorescence staining

Cells were harvested on day 2 post-transfection for analysis, washed once with PBS and fixed with 4% paraformaldehyde in PBS for 20 minutes at 4C. After blocked with 10% goat serum (Dako, Glostrap, Denmark), the cells were incubated with monoclonal mouse anti-SATB1 (1:50 dilution; Sigma, St Louis, MO) for 2 hours at 37C. After three washes, the cells were incubated with Cy3-conjugated rabbit anti-mouse secondary antibodies (1:300 dilution; ICN Cappel, USA) for 1 hour at 37C and washed three times with PBS. The stained cells were mounted and analyzed under fluorescence microscope. DAPI was used to visualize nuclei.

### Measurement of cell growth

Cell proliferation was measured using the methyl thiazole tetrazolium (MTT) assay [24,25]. Cells were seeded in 24-well plates at a density of 110^4^ cells/well and 24 hours later 200l 5mg/l MTT (Sigma) in PBS was added to each well incubated for 4 hours at 37C and the precipitate was solubilized in 100l 100% dimethylsulfoxide (Sigma) with shaking for 15 minutes. Absorbance values were determined using an enzyme-linked immunosorbent assay reader (Model 318, Shanghai, China) at 540nm. Each assay was performed nine times and the results are expressed as the meanSE compared to the control.

### Measurement of apoptosis by flow cytometry

U251 cells were harvested on hour 24 and 48 post-transfection for analysis, After washing with PBS fixed in 70% cold ethanol treated with 10g/L RNase suspended and stained with 10g/L propidium iodine, U251 cells were stained directly with PI at a concentration of 10g/ml and 2% Annexin-V-Fluos (Roche, Basel, Swizerland) in incubation buffer for 10 minutes. Cells were acquired with the FACS calibrator (BD) after setting the instrument with the controls (nontreated, stained cells), after two washes in PBS. In this experiment, cells with early apoptotic signals, stained with annexin-V, and cells with late death signals, stained with PI, were considered and quantified, and the apoptotic cells were analyzed using CellQuest software. Each assay was performed in triplicate.

### Tumor cell adherence to ECV304

ECV304 cells were plated in 96 well plates at a density of 510^4^ cells/well cultured for 48 hours, the supernatant was aspirated and untransfected U251, control-shRNA-GFP U251 or SATB1-shRNA U251 cells were plated at a density of 510^4^ cells/well and cultured for 30 minutes. The wells were washed twice with PBS to remove unattached cells 100l 25% rose Bengal solution was added, incubated for 5 minutes, the supernatant was aspirated, the wells were washed twice with PBS. 200l 95% ethanol/PBS (1:1) was added, incubated for 20 minutes and absorbance was measured at 540nm. Each assay was performed in triplicate.

### Adhesion assay

Cells were seeded in quadruplicate at a density of 110^4^ cells/well in 96 well plates coated with 10g/L BSA, 50mg/L Matrigel, or 10mg/L fibronectin (Fn), cultured at 37C for 60 minutes, and the MTT assay was performed as previously described [26,27]. Each assay was performed in triplicate.

### Invasion assay

The invasion assays with cells were performed using Transwell polycarbonate membrane inserts in 24-well plates (Corning, Lowell, MA) following the manufacturers instructions. Briefly, the underside of each polycarbonate microporous membrane was coated with Matrigel (1:100) at 37C for 5 minutes and allowed to sit overnight. Then, 50l Matrigel (1:30) and 200l sterile water were added to the upper compartment at 37C. After 2days, 200l of the invasion buffer [2ml BSA (2%)+38ml RPMI 1640] was added into the upper compartment and, 1 hour later, the upper compartment fluid was aspirated. Cells at a density of 510^4^ cells/well were added into the upper compartment, and 800l of the Fn solution (10g/ml) was added into the lower compartment. The cells were allowed to migrate for 48 hours. The inserts were then fixed in 10% formalin, stained with hematoxylin and eosin, and rinsed by dipping in water. The cells on the upper surface of the membrane were removed with a cotton bud. The membranes were air-dried overnight, excised from the insert, and mounted onto glass slides for microscopic analysis. The migrated cells were counted at high-power magnification (40) from four randomly selected fields. Each experiment was repeated three times.

### *In vitro* angiogenesis assay

The test was performed using the *In vitro* Angiogenesis Assay Kit (Chemicon International, Temecula, CA) following the manufacturers instructions. Briefly, 96-well plates were coated with cold solution (50l/well of a solution containing 900l of ECMatrix per 100l of 10 diluent buffer), which was allowed to polymerize at room temperature for about 60 minutes. Then, wells were seeded with 100l of a 510^4^ cells/ml suspension of ECV304, ECV304 transiently transfected with pHK, or ECV304 transiently transfected with SATB1-shRNA. Tube formation was assessed after 12 hours.

### Murine xenograft model

Male 4 to 6week old BALB/c athymic nude mice were subcutaneously injected with 210^6^ untransfected U251, control-shRNA-GFP U251 or SATB1-shRNA U251 cells. Tumor diameters were measured at regular intervals with digital calipers, and the tumor volume in mm^3^ was calculated using the formula: volume=(width)^2^length/2. The animal experiments in this study were performed in compliance with the guidelines of the Institute for Medical School Institutes at Wuhan University and Shanghai Jiao Tong University.

### Data analysis

Statistical analyses and graphs were performed using the Statistical Package for the Social Sciences (version 12.0, for Windows) (SPSS, Chicago, IL, USA). Quantitative values were expressed as meanSD. Statistical differences between groups were examined using the Fisher's exact test. *P*-values less than 0.05 were considered statistically significant.

## Results

### Immunohistochemical analysis of SATB1 expression in human glioma and normal brain tissue

**Figure 1:**
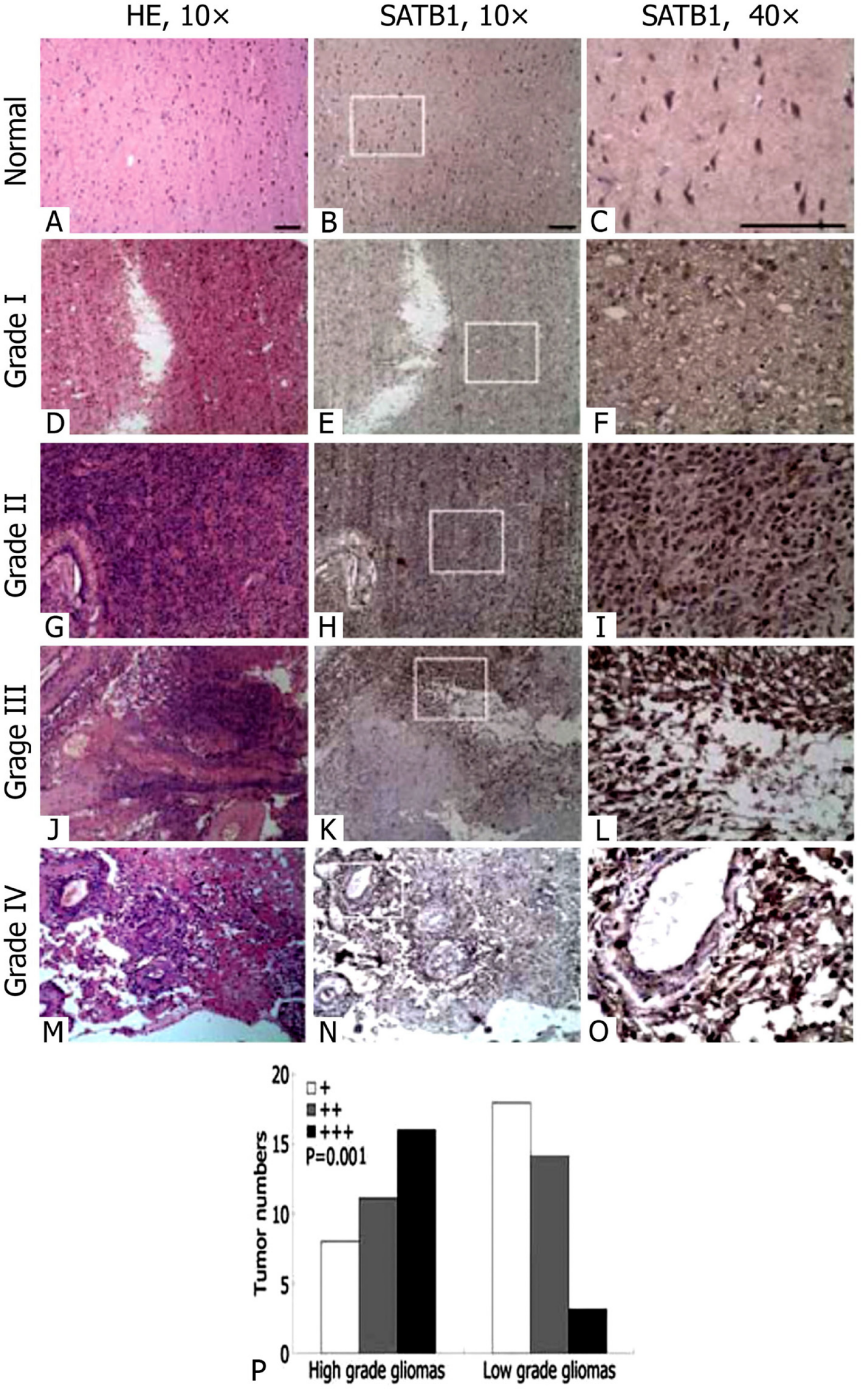
**Immunohistochemical staining of SATB1 expression in human glioma and normal cerebral cortex tissue.A**-**C**) normal cerebral cortex tissue; **D**-**F**) glioma with WHO grade I; **G**-**I**) glioma with WHO grade II; **J**-**L**) glioma with WHO grade III; and **M**-**O**) glioma with WHO grade IV. Serial sections of the same samples were used for hematoxylin and eosin (HE) staining. Magnified40 panels represented the white rectangles in the10 panels. Scale bar=100m. **P**) Semiquantitative analysis of SATB1 expression in high grade gliomas and low grade gliomas, *P* value compares overall SATB1 expression in each group.

We examined the expression of SATB1 in 70 gliomas and the normal brain tissues using immunohistochemistry. The low expression of SATB1 were found in the normal brain tissues (Figure 1A-C). In glioma tissues, brown positive staining was mostly homogeneously distributed within the nucleolus, and in the high grade glioma tissues, SATB1 was expressed at increased levels compared to the low grade glioma tissues (Figure 1D-O). Semi-quantitative analysis indicated a significant increase in SATB1 expression in high grade gliomas and low grade gliomas (*P*=0.001, Figure 1P). The percentage of glioma tissues that exhibited positive staining of SATB1 was 62.9%.

### Expression of SATB1 in human glioma and normal brain tissues as determined by RT-PCR and western blotting

**Figure 2:**
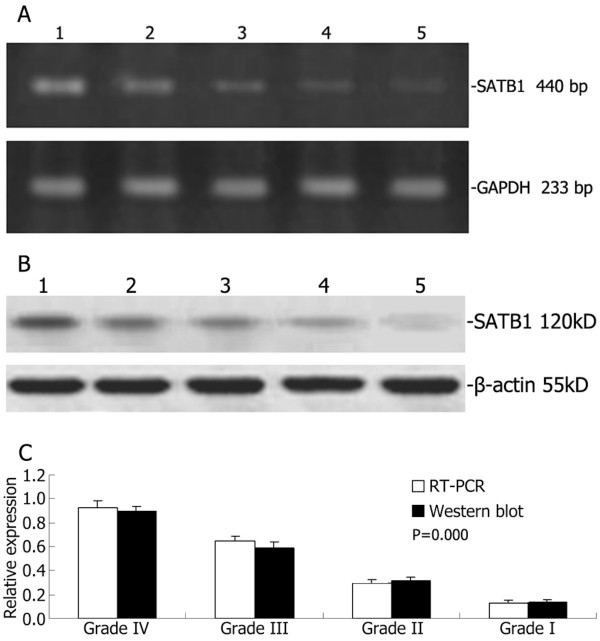
**RT-PCR and Western blotting analysis of SATB1 expression in human glioma and normal brain tissue.** Representative images of SATB1 RT-PCR (**A**) and Western blot (**B**). Lane 1, glioma with WHO grade IV; lane 2, glioma with WHO grade III; lane 3, glioma with WHO grade II; lane 4, glioma with WHO grade I; lane 5, normal brain tissue. (**C**) The ratio of SATB1 mRNA/protein expression to glyceraldehyde 3-phosphate dehydrogenase (GADPH)/-actin showing increased SATB1 mRNA/protein expression in high grade gliomas compared to low grade gliomas.

The low expression of SATB1 mRNA and protein were found in the three normal brain tissues and the expression of SATB1 mRNA and protein was increased in the high grade glioma samples compared with the low grade glioma tissues (Figure 2A-B). Furthermore, the RT-PCR and western blotting analysis showed that the ratio of the high grade glioma tissues was more than that of low grade glioma tissues (Figure 2C).

### Relationship between SATB1 expression, clinicopathologic characteristics and MGMT promoter methylation

Correlations between the expression of SATB1 and various clinicopathologic parameters and between the expression of SATB1 and MGMT promoter methylation were listed in Table 1. The expression of SATB1 was significantly related to the pathological grade of glioma (*P*=0.025). Overexpression of SATB1 was associated with high pathological grade (WHO III-IV). The expression of SATB1 was significantly related to MGMT promoter methylation (*P*=0.000). However, no statistically significant differences were identified between SATB1 expression in relation to age, sex, position or tumor size.

**Table 1:** Correlations between SATB1 expression, clinicopathologic features and MGMT promoter methylation in 70 cases of glioma

**Clinicopathologic variables**	**n**	**SATB1 expression**		***X***^2^	***P*-value**
		**Negative**	**Positive**		
All cases	70	26	44		
Age (years)				1.004	0.446
<45	27	12	15		
45	43	14	29		
Gender				0.388	0.566
Male	54	19	35		
Female	16	7	9		
Tumor size (cm)				0.227	0.795
<3	24	8	16		
3	46	18	28		
Tumor locus				0.946	0.393
Supratentorial	53	18	35		
Infratentorial	17	8	9		
Pathological grade				6.119	0.025
Low grade I-II	35	18	17		
High grade III-IV	35	8	27		
MGMT promoter				25.150	0.000
Unmethylation	45	7	38		
Methylation	25	19	6		

### Univariate and multivariate analyses of prognostic variables in patients with glioma

**Figure 3:**
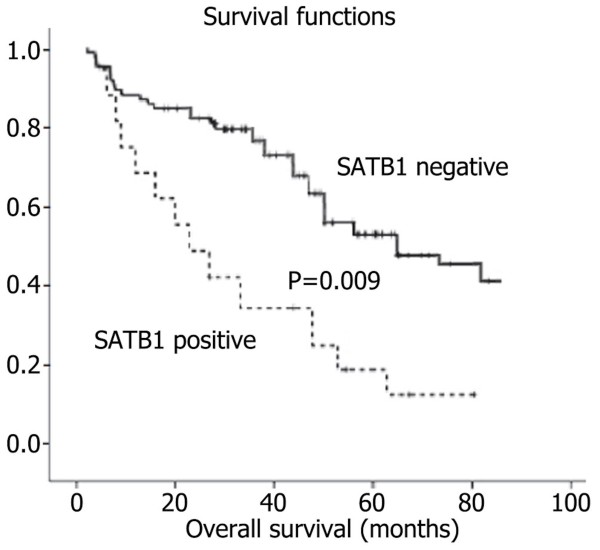
**Kaplan-Meier survival analysis of glioma patients after surgical resection.** Samples with positive SATB1 expression (n=44) and negative SATB1 expression (n=26) were analyzed. The survival rate for patients in the SATB1 positive group (+) was significantly lower than that for patients in the SLC22A18 negative group () (log rank, *P*=0.009).

The 5-year overall survival rates of patients with positive and negative SATB1 expression were 18.2% (8/44) and 53.8% (14/26) respectively, and there was significant difference in 5-year overall survival rates (*P*=0.002). The 5-year survival rates of patients with positive and negative SATB1 expression in high grade glioma were 0/27 and 2/8 respectively, and there was significant difference in 5-year survival rates (*P*=0.007). The 5-year survival rates of patients with positive and negative SATB1 expression in low grade glioma were 8/17 and 12/18 respectively, and there was no significant difference in 5-year survival rates (*P*=0.241). Thus, future studies with larger sample sizes should be done to confirm this trend. Patients showing positive SATB1 expression in high grade glioma had a significantly shorter overall survival period than those with negative expression (*P*=0.009, log-rank test; Figure 3). Univariate Cox regression analysis also identified that clinical variables including the pathological grade of glioma, SATB1 expression and MGMT promoter methylation were significantly associated with overall survival (Table 2). Furthermore, multivariate Cox regression analysis (Forward: LR) was performed to evaluate the potential of SATB1 expression as an independent predictor for overall survival of glioma patients. While other factors failed to demonstrate independence, the correlation between the pathological grade of glioma, SATB1 expression and MGMT promoter methylation may play a role in predicting overall survival in glioma (*P*=0.046 and *P*=0.015, *P*=0.012, respectively, Table 2).

**Table 2:** Univariate and multivariate analyses of different prognostic factors in patients with gliomas

**Variables**	**Relative risk (95% CI)**	***P*-value**
Univariate		
Age	0.794 (0.413-1.275)	0.253
Sex	0.715 (0.362-1.389)	0.342
Tumor size	0.932 (0.579-2.486)	0.356
Tumor locus	0.683 (0.362-1.142)	0.182
Pathological grade	1.854 (1.143-3.045)	0.035
SATB1	0.448 (0.229-0.788)	0.009
MGMT methylation	0.224 (0.102-0.356)	0.004
Multivariate		
Pathological grade	0.654 (0.453-1.026)	0.046
SATB1	0.446 (0.248-0.792)	0.015
MGMT methylation	0.412 (0.218-0.681)	0.012

### Association of the Ki67 index with SATB1 expression in glioma

Ki67 immunostainings were widely variable in different pathological grade gliomas and Ki67 were intensely expressed in cell nuclei of glioma. The Ki67 indexes of glioma tissues with positive SATB1 expression were (49.124.26)%, which were significantly higher than that of glioma tissues without detectable SATB1 expression (Table 3).

**Table 3:** Association between Ki67 index and SATB1 expression in glioma tissues

**SATB1**	**n**	**Ki67 index (%, meanSD)**	***P***
Positive	44	49.124.26	0.000
Negative	26	10.346.52	

### SATB1, c-Met, SLC22A18, caspase-3 and bcl-2 expression in U251 cells and cell proliferation assay

The SATB1 mRNA and protein inhibition rate of SATB1-shRNA U251 cells was 92% and 86% compared with the untransfected U251 cells respectively, whereas the control-shRNA-GFP U251 cells had not such change (see Additional file 1: Figure S1A-D). The SATB1 protein inhibition rate of SATB1-shRNA U251 cells was 83% compared with the untransfected U251 cells by immunofluoresence staining, whereas the control-shRNA-GFP U251 cells had not such change (see Additional file 2: Figure S2A and B). SATB1 was stained red and located in nuclei of cells. The c-Met protein inhibition rate of SATB1-shRNA U251 cells was 61% compared with the untransfected U251 cells, whereas the control-shRNA-GFP U251 cells had not such change (see Additional file 3: Figure S3A-B). Each group revealed that in SATB1-shRNA U251 cells the expression of SLC22A18 and caspase-3 increased, whereas the expression of bcl-2 decreased (see Additional file 3: Figure S3A-B). SATB1-shRNA caused a statistically significant reduction of cell viability to (29.57.24)%, whereas the control-shRNA-GFP U251 cells had not such change (see Additional 4: Figure S4).

### Induction of apoptosis by SATB1-shRNA

**Figure 4:**
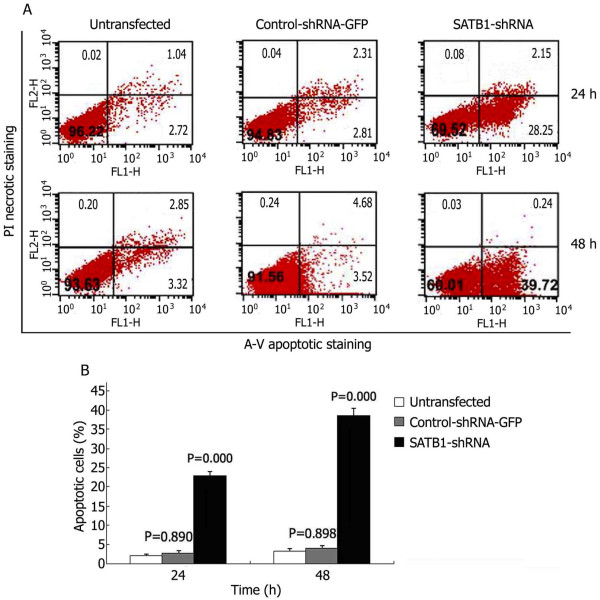
**FACS Analysis of Annexin-V staining of U251 cells after transfection.** (**A**) Representative FACS scatter plots of U251 cells. (**B**) Percentages of apoptotic cells in U251 cells after transfection.

To quantitate the SATB1-shRNA induced apoptotic cell death in U251 cells, approximately 1 10^6^ U251 cells were double stained with Annexin-V-FITC and propidium iodide (PI) at different times post transfection. Apoptotic cell death was detected from 24 hours and 48 hours after transfection (Figure 4A). FACS analysis identified significantly higher numbers of apoptotic cells in SATB1-shRNA transfected U251 cells than untransfected control cells (Figure 4B).

#### Effects of SATB1-shRNA on U251 cell adhesion

**Figure 5:**
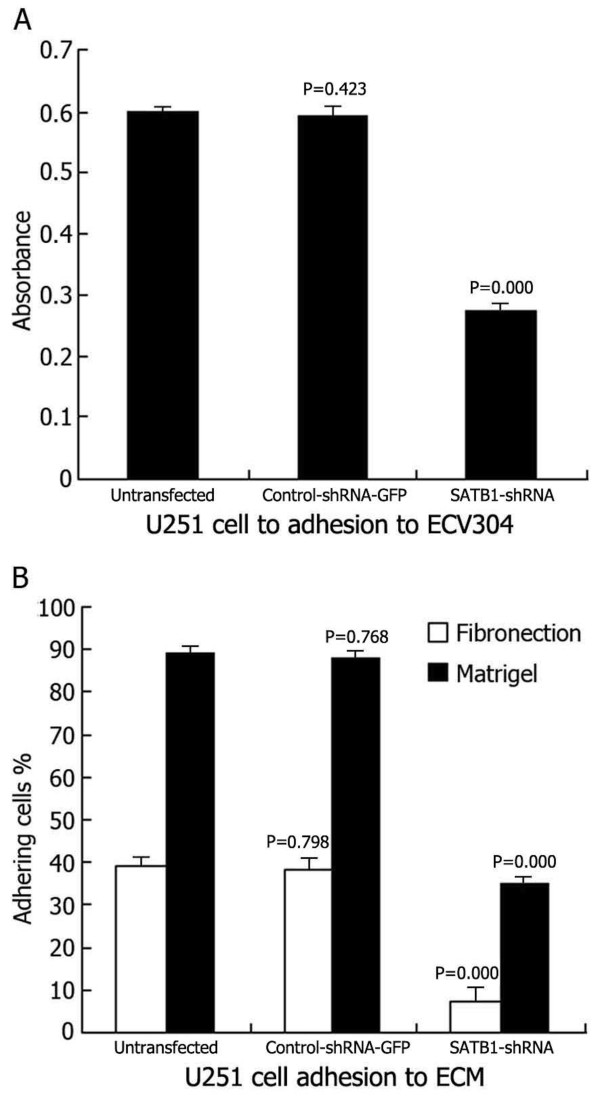
**Effects of SATB1-shRNA on U251 cell adhesion.** (**A**) U251 cells adhesion to ECV304. (**B**) U251 cells adhesion to ECM (Fn and Matrigel).

The tumor cell lines showed different absorbance abilities: untransfected U251 cells, 0.6020.007; control-shRNA-GFP U251 cells, 0.5930.016; SATB1-shRNA U251 cells, 0.2620.014 (Figure 5A). Suppressing SATB1 expression had a clear inhibitory effect on the adhesion of transfected U251 cells to the extracellular matrix (ECM) [Matrigel and Fn] and to ECV304. The percentages of adhesion to ECM were as follows: untransfected U251 cells, (39.52.24)% (Fn) and (90.21.54)% (Matrigel); control-shRNA-GFP U251 cells, (38.93.08)% (Fn) and (89.81.56)% (Matrigel); SATB1-shRNA U251 cells, (7.93.25)% (Fn) and (36.21.62)% (Matrigel) (Figure 5B). Thus, the adhesion of U251-SLC22A18 to ECV304 and to ECM cells was significantly suppressed.

#### Effects of SATB1-shRNA on U251 cell invasion

**Figure 6:**
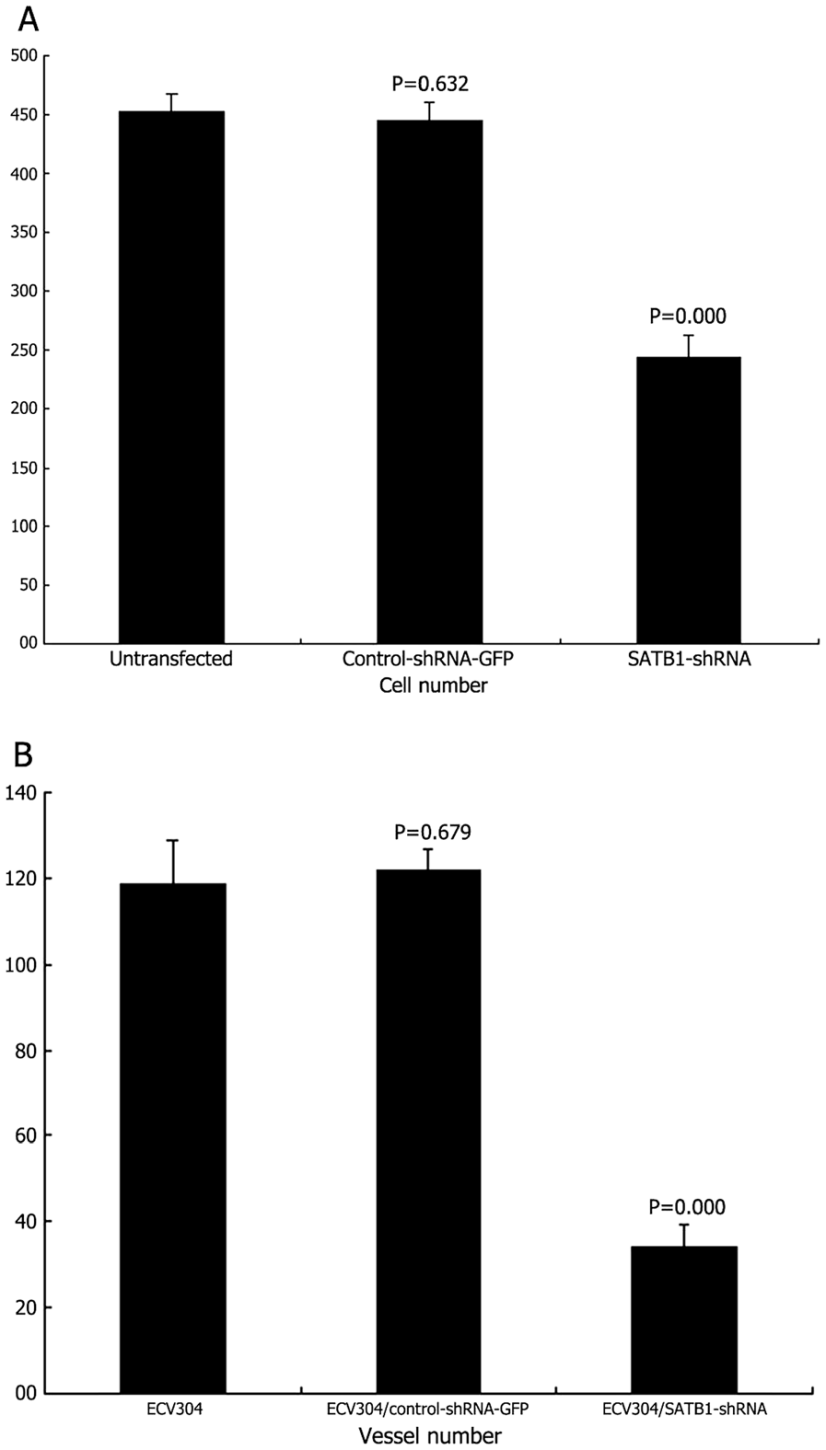
**Effects of SATB1-shRNA on U251 cell invasion and angiogenesis *in vitro*.** (**A**) U251 cell invasion. (**B**) Angiogenesis *in vitro*.

As shown in Figure 6A, for each 400 field under the microscope, the number of migrated SATB1-shRNA U251 cells was 24325, significantly lower than the number of untransfected U251 cells (45218) and the control-shRNA-GFP U251 cells (44515). In addition, there was little difference between untransfected U251 cells and the control-shRNA-GFP U251 cells.

#### Effects of SATB1-shRNA on angiogenesis *in vitro*

As shown in Figure 6B, *in vitro* tube formation of ECV304 cells transiently transfected with SATB1-shRNA was 345 per 100 field, which was significantly lower compared with untransfected ECV304 (11910) and ECV304 transiently transfected with control-shRNA-GFP (1226). Moreover, there was little difference between untransfected ECV304 and ECV304 transiently transfected with control-shRNA-GFP.

#### Effects of the SATB1-shRNA on tumor growth *in vivo*

**Figure 7:**
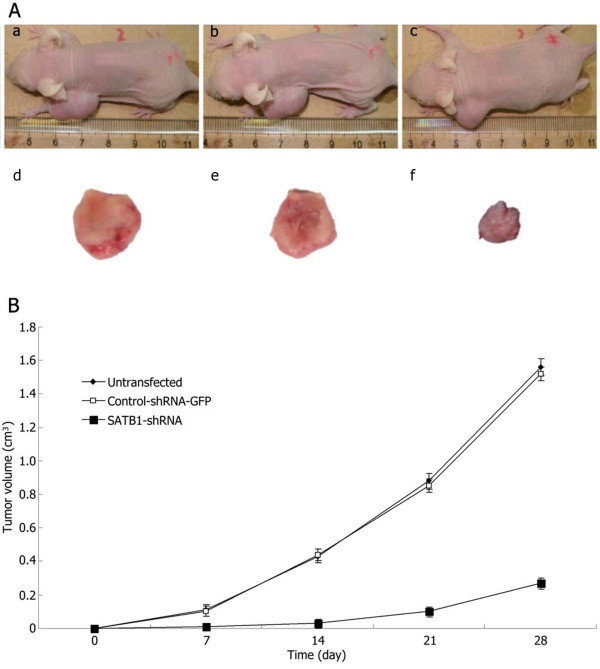
**Effects of SATB1-shRNA on tumor growth *in vivo*.** (**A**) Subcutaneous tumor model. a and d, untransfected U251 cells group; b and e, control-shRNA-GFP U251 cells group; c and f, SATB1-shRNA U251 cells group. (**B**) Tumor growth curves of each group over 28days.

As shown in Figure 7, untransfected U251 and control-shRNA-GFP U251 xenograft tumors formed and grew rapidly. In contrast, SATB1-shRNA U251 xenograft tumor formation was significantly delayed. At the end of the experiment, the SATB1-shRNA U251 tumors were significantly smaller than the tumors from untransfected U251 and control-shRNA-GFP U251 cells.

#### Effects of the SATB1-shRNA on tumor angiogenesis *in vivo*

**Figure 8:**
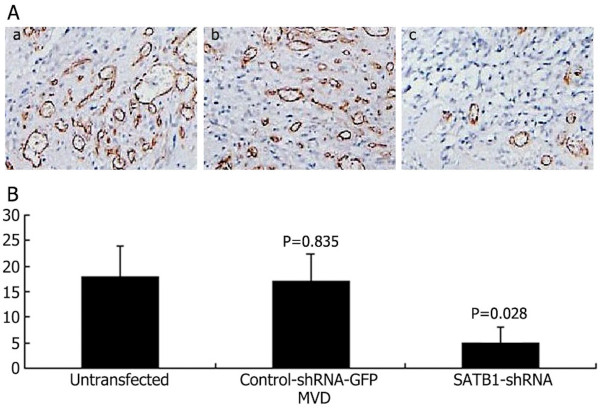
**Effects of SATB1-shRNA on tumor angiogenesis *in vivo*.** (**A**) Expression of CD34 in subcutaneous tumors. a, untransfected U251 cells group; b, control-shRNA-GFP U251 cells group; c, SATB1-shRNA U251 cells group. (**B**) The MVD values (per 200 field) of subcutaneous tumors.

Tumor tissue from mice was excised and subjected to immunohistochemical staining. As shown in Figure 8, the microvascular density (MVD) values (per 200 field) of subcutaneous tumors in untransfected U251, control-shRNA-GFP U251, and SATB1-shRNA U251 cells were 186, 175, and 53, respectively. These results indicate that CD34-positive vessels were abundant in subcutaneous tumors in the untransfected U251, control-shRNA-GFP U251 cells, whereas vessel density in both tumor types was significantly decreased in the SATB1-shRNA U251 cells group.

## Discussion

Glioma is one of the most aggressive human tumors and the prognosis for glioma patients is bleak, even with improved diagnosis and composite therapy [28]. Therefore, identification of prognostic molecular biomarkers is invaluable for the clinician to evaluate patients and to aid in tumor control, and studies of the underlying molecular mechanisms involved in glioma formation and progression provide tremendous opportunities to identify molecules which may provide novel potential drug design targets for the treatment of brain tumors. Using molecular analysis, loss of heterozygosity has been observed on several chromosomes in patients with glioma. Many of these chromosomal segments contain known tumor suppressor genes [26,29], such as p53 on 17p and *SLC22A18* on 11p15.5. Mutations and overexpression of several genes, including c-Met, PDGF and c-myc, have been identified in glioma patients [30,31].

SATB1 is a tissue-specific nuclear matrix-attachment-DNA-binding protein, which is located on chromosome 3p23. The investigations of SATB1 were carried out mainly in immune cells in the past. SATB1 plays an important role in the development and maturation of CD8SP T cells [9]. It is a notable organizer of thymocyte chromatin which controls gene expression and gene recombination in developing thymocytes [32]. SATB1 has recently attracted considerable attention due to its high expression in tumor tissues of a variety of malignancies [9-13], which suggest a crucial role in promoting tumor growth, invasion and metastasis, and may also have a potential value of being a candidate for cancer therapy [14]. SATB1 can regulate the expression of other genes by many mechanisms [8,33-36]. Recently, Han *et al.*[15]. have discovered that SATB1 protein is expressed in poorly differentiated infiltrating tumor, but is absent in normal tissue. 1,318 breast cancer specimens were analyzed in the study and revealed a correlation between higher SATB1 expression levels and shorter overall survival times. They also revealed that a knockdown of SATB1 in highly aggressive (MDA-MB-231) cancer cells altered the expression of 1,000 genes, reversing tumorigenesis and inhibiting tumor growth and metastasis *in vivo*. These observations indicate that SATB1 reprograms chromatin organization and the transcription profiles of tumors to promote growth, spreading and metastasis; however, SATB1 expression in glioma and the relationship with glioma progression has not previously been investigated in humans.

In our study, we demonstrated that of 70 tumors, 44 (62.9%) were positive for SATB1 expression. The 5-year overall survival rates of patients with positive and negative SATB1 expression were 18.2% (8/44) and 53.8% (14/26) respectively, and there was significant difference in 5-year overall survival rates. The 5-year survival rates of patients with positive and negative SATB1 expression in high grade glioma were 0/27 and 2/6 respectively, and there was significant difference in 5-year survival rates. Our results showed that SATB1 expression was significantly associated with a high histological grade and with poor survival in univariate and multivariate analyses. Correlation analysis showed the expression of SATB1 is correlated with MGMT promoter methylation which is a key prognostic factor and can predict treatment response in glioma [37,38]. In our Cox regression analysis, MGMT promoter methylation was considered as an independent prognostic factor. Cox multivariable analysis showed that SATB1 expression correlated with poor prognosis in patients with gliomas and was an independent prognostic factor. SATB1 expression was also positively correlated with Ki67 expression in glioma tissue. SATB1 shRNA expression vectors could efficiently induce the expression of SLC22A18 protein and increase the caspase-3 protein, and inhibit the expression of SATB1, c-Met and bcl-2 protein and the growth, invasion, metastasis and angiogenesis of U251 cells and, induced apoptosis *in vitro*. Furthermore, the tumor growth of U251 cells expressing SATB1 shRNA were inhibited *in vivo*, and immunohistochemical analyses of tumor sections revealed a decreased vessel density in the animals where shRNA against SATB1 were expressed. Our results revealed that a knockdown of SATB1 in highly aggressive glioma U251 cells could alter the expression of c-Met, SLC22A18, caspase-3 and bcl-2 protein, reversing tumorigenesis, inhibiting tumor growth, invasion and angiogenesis, and inducing apoptosis. These observations indicate that SATB1 may reprogram chromatin organization and the transcription profiles of tumors to promote growth and invasion. These results indicated that SATB1 may have an important role as a positive regulator of glioma development and progression.

## Conclusions

Collectively, our findings indicated for the first time that SATB1 was overexpressed in human glioma, and SATB1 expression in human glioma was associated with clinicopathological factors and prognosis. The high SATB1 expression, directly contributing to tumor development and progression, might be a candidate independent prognostic marker for predicting the outcome of human glioma. Furthermore, the present study demonstrated that RNA interference of SATB1 successfully inhibited the expression of SATB1 protein and mRNA, the growth, adhesion, invasion, metastasis and angiogenesis *in vitro* and tumor growth and angiogenesis *in vivo*, which might be the result of reducing expression of SATB1, c-Met and bcl-2 protein and increasing the expression of SLC22A18 and caspase-3.

## Abbreviations

SATB1: Special AT-rich sequence-binding protein 1; MGMT: O(6) -methylguanine-DNA-methyltransferase; SLC22A18: Solute carrier family 22 (organic cation transporter) member 18; c-Met: Mepatocyte growth factor receptor; RT-PCR: Reverse transcription polymerase chain reaction; RPMI: Roswell park memorial institute; PBS: Phosphate-buffered saline; SDS: Sodium dodecyl sulfate; MSP: Methylation-specific polymerase chain reaction.

## Competing interests

The authors declare that they have no competing interests.

## Authors' contributions

SHC, YBM and DFF carried out the laboratory analysis. SHC, HZ and ZAZ participated in the design of the study and drafted the manuscript. ZQL and PCJ conceived of the study, and participated in its design and coordination and helped to draft the manuscript. All authors read and approved the final manuscript.

## Supplementary Material

Additional file 1 **Additional file 1: Figure S1.** RT-PCR and Western blotting analysis of SATB1 expression inhibited by shRNA and the inhibition rate. Representative images of SATB1 RT-PCR (A) and Western blot (C). The SATB1 mRNA expression inhibition rate of SATB1-shRNA (B) and the SATB1 protein expression inhibition rate of SATB1-shRNA (D) in U251 cells. Lane 1, untransfected U251 cells; lane 2, control-shRNA-GFP U251 cells; lane 3, SATB1-shRNA U251 cells. Click here for file

Additional file 2 **Additional file 2: Figure S2.** Immunofluoresence staining analysis of SATB1 expression inhibited by shRNA and the inhibition rate. Representative images of the untransfected U251 cells (A), control-shRNA-GFP U251 cells (B), SATB1-shRNA U251 cells (C), and the inhibition rate of untransfected U251 cells, control-shRNA-GFP U251 cells and SATB1-shRNA U251 cells (D). Nuclei were counterstained using DAPI. Scale bar = 25 m. Click here for file

Additional file 3 **Additional file 3: Figure S3.** Western blotting analysis of c-Met, SLC22A18, caspase-3 and bcl-2 protein expression. (A) Representative images of western blotting analysis of c-Met, SLC22A18, caspase-3 and bcl-2 expression. (B) Level of the c-Met, SLC22A18, caspase-3 and bcl-2 protein expression in U251 cells. Lane 1, untransfected U251 cells; lane 2, control-shRNA-GFP U251 cells; lane 3, SATB1-shRNA U251 cells. Click here for file

Additional file 4 **Additional file 4: Figure S4.** Cytotoxic effect of SATB1-shRNA in U251 cells. The untransfected U251 cells, control-shRNA-GFP U251 cells and SATB1-shRNA U251 cells were cultured in plastic 96-well plates and quantified using the MTT assay. Click here for file
