# Supplementary figures and images for: Upregulation of SATB1 is associated with the development and progression of glioma
Source: J Transl Med. 2012 Jul 28;10:149. doi: 10.1186/1479-5876-10-149 (PMC3492129; doi:10.1186/1479-5876-10-149)

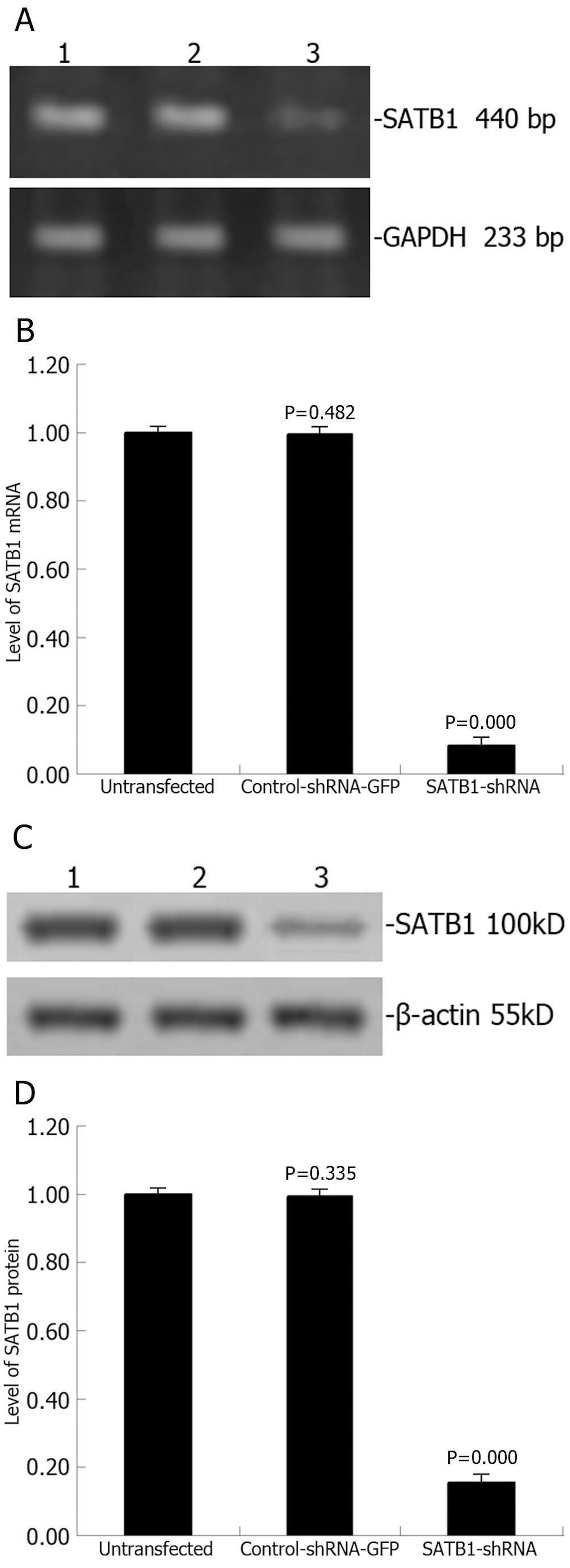

Supplement: Additional file 1 — Figure S1. RT-PCR and Western blotting analysis of SATB1 expression inhibited by shRNA and the inhibition rate. Representative images of SATB1 RT-PCR (A) and Western blot (C). The SATB1 mRNA expression inhibition rate of SATB1-shRNA (B) and the SATB1 protein expression inhibition rate of SATB1-shRNA (D) in U251 cells. Lane 1, untransfected U251 cells; lane 2, control-shRNA-GFP U251 cells; lane 3, SATB1-shRNA U251 cells. [file 1479-5876-10-149-S1.tiff]

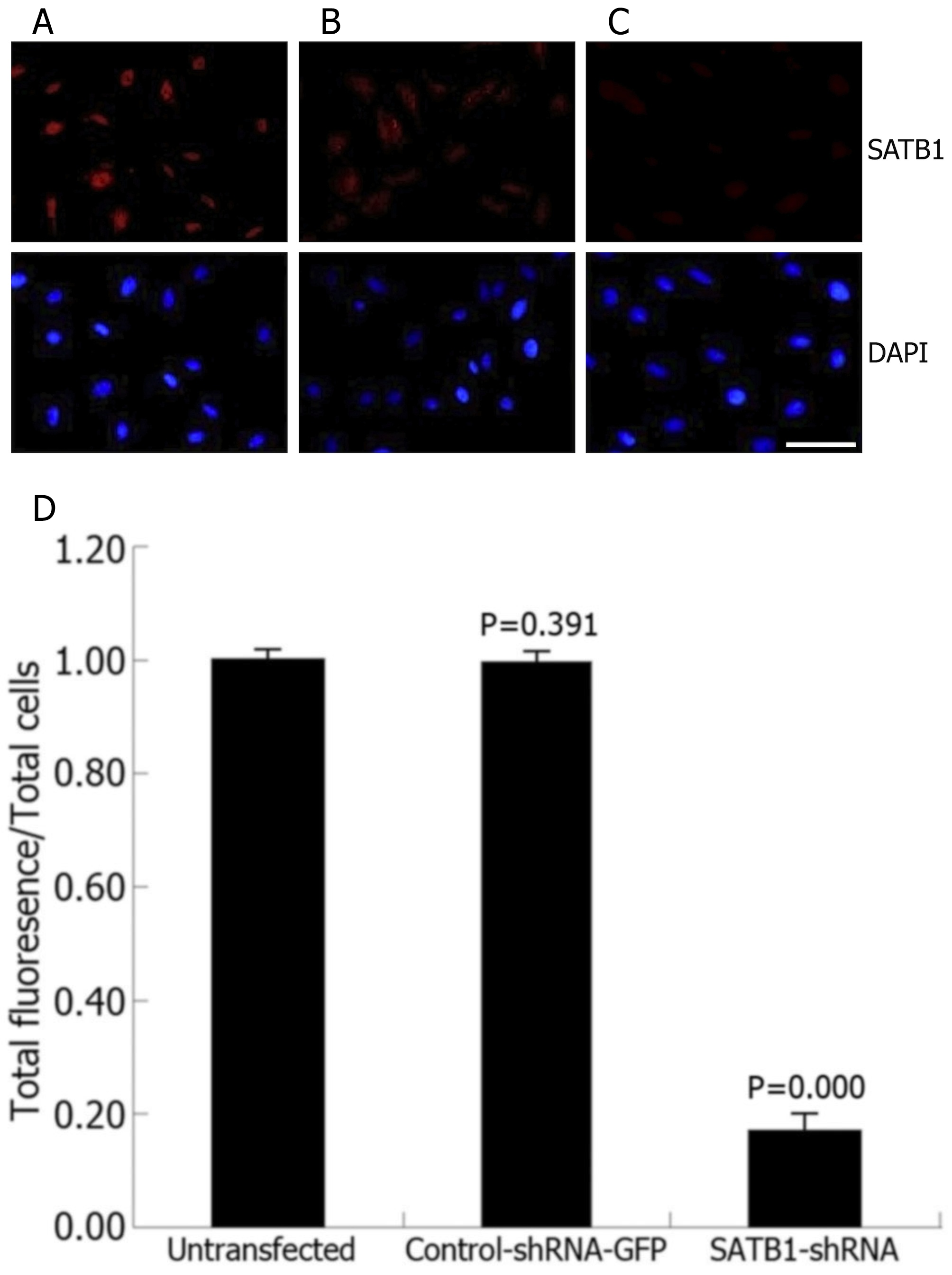

Supplement: Additional file 2 — Figure S2. Immunofluoresence staining analysis of SATB1 expression inhibited by shRNA and the inhibition rate. Representative images of the untransfected U251 cells (A), control-shRNA-GFP U251 cells (B), SATB1-shRNA U251 cells (C), and the inhibition rate of untransfected U251 cells, control-shRNA-GFP U251 cells and SATB1-shRNA U251 cells (D). Nuclei were counterstained using DAPI. Scale bar = 25 m. [file 1479-5876-10-149-S2.tiff]

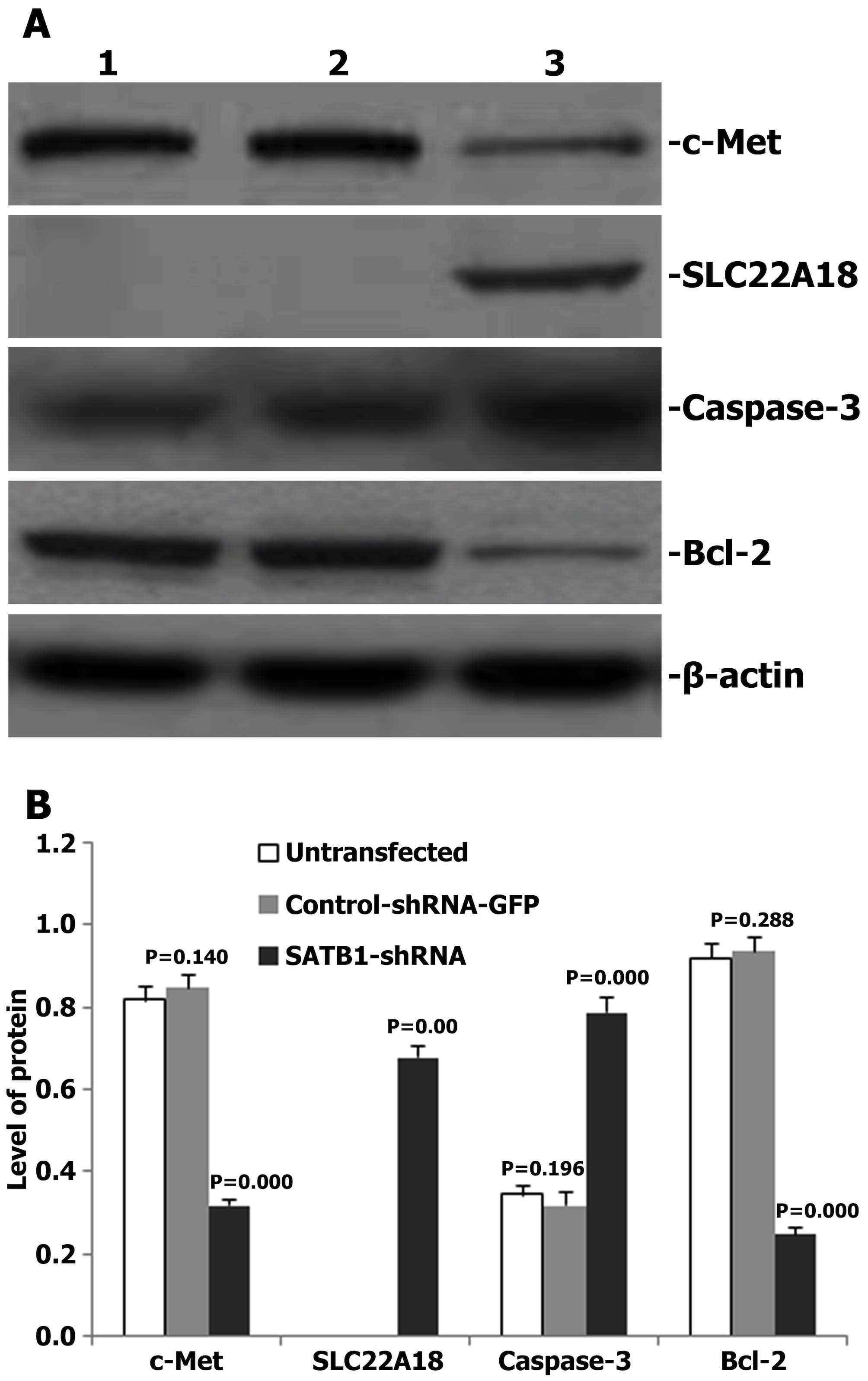

Supplement: Additional file 3 — Figure S3. Western blotting analysis of c-Met, SLC22A18, caspase-3 and bcl-2 protein expression. (A) Representative images of western blotting analysis of c-Met, SLC22A18, caspase-3 and bcl-2 expression. (B) Level of the c-Met, SLC22A18, caspase-3 and bcl-2 protein expression in U251 cells. Lane 1, untransfected U251 cells; lane 2, control-shRNA-GFP U251 cells; lane 3, SATB1-shRNA U251 cells. [file 1479-5876-10-149-S3.tiff]

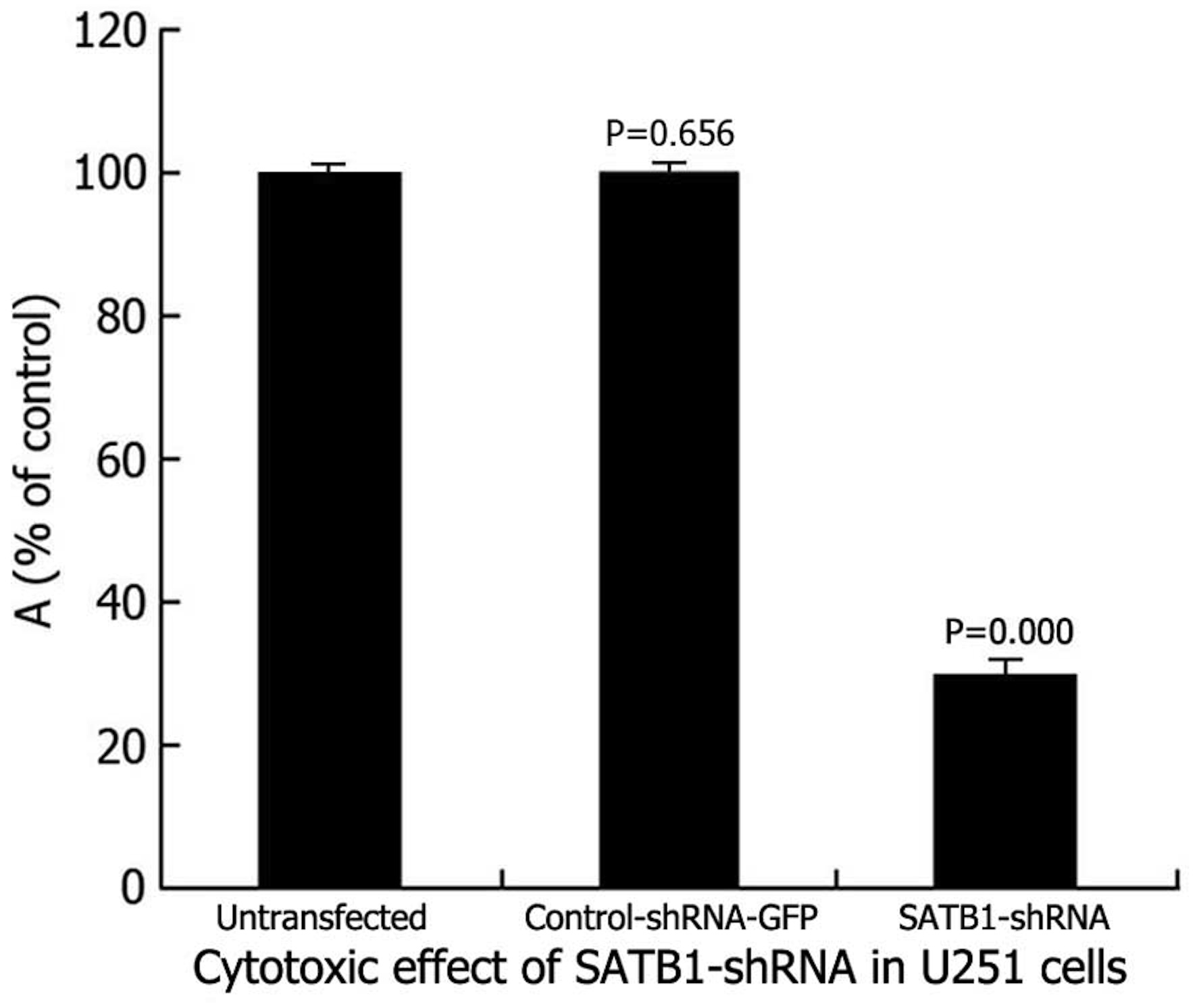

Supplement: Additional file 4 — Figure S4. Cytotoxic effect of SATB1-shRNA in U251 cells. The untransfected U251 cells, control-shRNA-GFP U251 cells and SATB1-shRNA U251 cells were cultured in plastic 96-well plates and quantified using the MTT assay. [file 1479-5876-10-149-S4.tiff]
